# Identifying and Mitigating Phishing Attack Threats in IoT Use Cases Using a Threat Modelling Approach

**DOI:** 10.3390/s21144816

**Published:** 2021-07-14

**Authors:** Syed Ghazanfar Abbas, Ivan Vaccari, Faisal Hussain, Shahzaib Zahid, Ubaid Ullah Fayyaz, Ghalib A. Shah, Taimur Bakhshi, Enrico Cambiaso

**Affiliations:** 1 Al-Khwarizmi Institute of Computer Science (KICS), University of Engineering & Technology (UET), Lahore 54890, Pakistan; faisal.hussain@kics.edu.pk (F.H.); shahzaib.zahid@kics.edu.pk (S.Z.); ubaid.fayyaz@kics.edu.pk (U.U.F.); ghalib@kics.edu.pk (G.A.S.); 2 Consiglio Nazionale delle Ricerche (CNR), IEIIT Institute, 16149 Genoa, Italy; enrico.cambiaso@ieiit.cnr.it; 3 Department of Computer Science, National University of Computer and Emerging Sciences, Lahore 54000, Pakistan; taimur.bakhshi@nu.edu.pk

**Keywords:** internet of things, mitigation, threat modeling, cyber-threats, phishing, smart autonomous vehicular system, smart home, cyber-security

## Abstract

Internet of things (IoT) is a technology that enables our daily life objects to connect on the Internet and to send and receive data for a meaningful purpose. In recent years, IoT has led to many revolutions in almost every sector of our society. Nevertheless, security threats to IoT devices and networks are relentlessly disruptive, because of the proliferation of Internet technologies. Phishing is one of the most prevalent threats to all Internet users, in which attackers aim to fraudulently extract sensitive information of a user or system, using fictitious emails, websites, etc. With the rapid increase in IoT devices, attackers are targeting IoT devices such as security cameras, smart cars, etc., and perpetrating phishing attacks to gain control over such vulnerable devices for malicious purposes. In recent decades, such scams have been spreading, and they have become increasingly advanced over time. By following this trend, in this paper, we propose a threat modelling approach to identify and mitigate the cyber-threats that can cause phishing attacks. We considered two significant IoT use cases, i.e., smart autonomous vehicular system and smart home. The proposed work is carried out by applying the STRIDE threat modelling approach to both use cases, to disclose all the potential threats that may cause a phishing attack. The proposed threat modelling approach can support the IoT researchers, engineers, and IoT cyber-security policymakers in securing and protecting the potential threats in IoT devices and systems in the early design stages, to ensure the secure deployment of IoT devices in critical infrastructures.

## 1. Introduction

**Figure 1:**
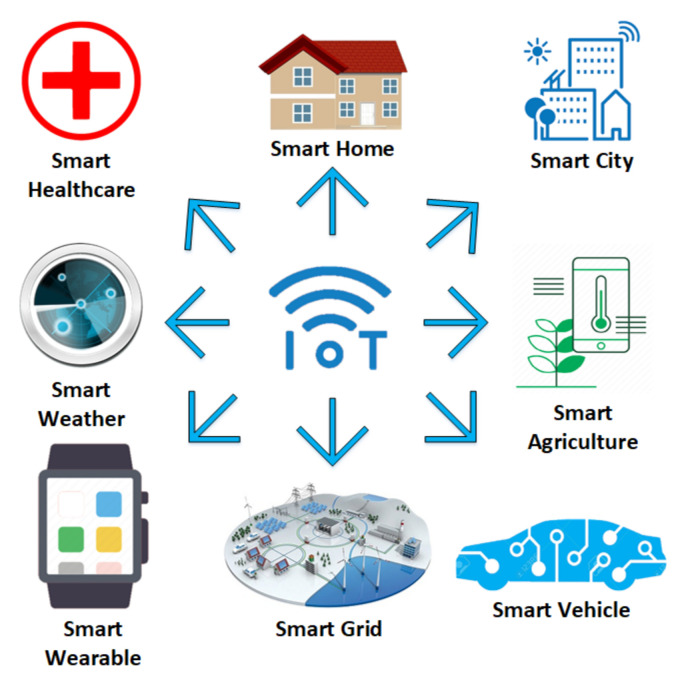
IoT Applications in Various Fields.

Internet of things (IoT) is a network of smart objects that can be connected through the Internet, to anonymously share data and resources. Primarily due to its non-interference with humans, larger extent, and extensible capabilities, IoT is superior to all other conventional networks [1]. The flourishing application of IoT has helped to devise many innovations, such as smart home, smart wearable, connected vehicles, smart grid, smart healthcare, and smart city [2]. Figure 1 illustrates some famous applications of IoT devices in various use cases in daily life.

Although the applications of IoT devices are rapidly evolving, the security of IoT devices is still a weakness of this technology [3]. Indeed, in recent years, the limited security measures led to several attacks on IoT devices. In addition, such attacks are increasing with the growth in available IoT devices [4]. This is because the manufacturers intensively focus on the production of IoT devices, without giving proper importance to the security perspective, due to increased consumer demand and high levels of competition from vendors [5]. The wide adoption of IoT devices by different organizations, industries, government sectors, etc., is at high risk due to the catastrophic effects of IoT device exploitation and data breaches. Hackers exploit the weaknesses of IoT devices, gain control over IoT devices and then perform malicious activities, such as botnet attacks, exposure of valuable and confidential information, etc., which causes financial loss [6].

Phishing is one of the top threats that cause data breaches [7,8,9]: it is a technique in which the attacker tries to steal a user’s credentials through fraudulent attempts, e.g., by sending emails [10]. The phishing phenomenon is increasing and becoming stronger, as phishers are increasing the sophistication of their techniques [11]. According to [12], many large companies such as Facebook, WhatsApp, UPS, Fargo, and Companies House (UK) experienced phishing attacks in recent years. In a data breach investigations report dated 2019, Verizon, a well-known US telecommunication company dealing in wireless products and services, revealed that phishing is the number one attack vector, as it was involved in 32% of the confirmed data breaches [9]. Even in 2020, phishing is reported as the most common cyber-threat [13].

Phishing attacks are performed using different communication mediums, such as email, website, instant messages, online social network, blogs, forms, and mobile applications [14]. As well as these phishing strategies, which use sensitive information about their intended targets, phishing emails are being increasingly tailored to look like real emails to increase the reaction time to the attacks. There has been an increase in email phishing and spear-phishing attacks in recent years, as these emails are designed to directly attack victim, with an increased likelihood of receiving a response [15].

These phishing attacks, including email phishing, are also performed using IoT devices [16,17]. These phishing attacks were performed by sending spam emails in a huge number three times a day [12,16]. As reported in [16,17], Proofpoint discovered that IoT devices, including smart TV, smart refrigerators, etc., have been used to send a bulk amount of spam emails. Due to the large number of internet-connected devices, up to 100,000 devices were used to send 750,000 malicious emails [12,16]. A total of 98% of IoT devices’ traffic is unencrypted and insecure against potential threats [18]. Therefore, the attackers can usually bypass the IoT devices’ traffic through phishing attacks and gather personal and sensitive information. Hence, a security mechanism is necessary to identify the threats, flaws, and countermeasures that may result in phishing attacks on IoT devices. Several tools have been developed to detect phishing attacks, such as Netcraft, AntiPhishing, and LinkExtend [19]. These tools are installed as extensions in web browsers, and phishing detection accuracy varies based on what type of email is received.

Although phishing is a well-investigated topic [14,20], research into the possibility of perpetrating phishing attacks in IoT contexts is still limited. These tools cannot be properly used in IoT devices to locate phishing attacks. The reason for this is that many IoT devices, such as smart door locks, smart wearables and smart sensors such as temperature sensors, do not provide a web interface to a user; instead, they are controlled via smartphone applications through Bluetooth [21]. Similarly, due to the resource-constrained nature—limited memory, limited computational power, limited storage, small battery, etc.—of IoT devices, most IoT devices do not allow a user to install and run on-device security applications to protect them from phishing or other cyber-attacks [22,23,24]. Consequently, these facts disclose that phishing identification in IoT is still a large problem after the development of phishing detection tools. There must be some approach that identifies the core reasons for and problems of the threats behind the phishing attacks. Threat modelling is an approach that helps us to identify the potential threats in a system [25]. From the identified threats, we can figure out which may cause phishing attacks.

Alot of work has been carried out to date to detect phishing attacks. However, the existing approaches detect phishing attacks after the attackers find a security loophole in an underlying end-device, and attempt to gain control over this using a phishing attack to perform malicious activities. Thus, there is a crucial need to protect the underlying device from phishing attacks by constricting the cyber-threats to null during the earlier design phase of a system design. Therefore, in this paper, we used a threat-modelling approach to identify security loopholes or threats during the design phase of a system. The proposed approach is very beneficial in preventing phishing attacks in IoT devices, as it identifies the threats in the earlier design stage of a system that may lead to phishing attacks after deployment. Moreover, we also proposed some mitigation strategies that the IoT security experts and IoT device vendors could consider while designing an IoT system to protect the underlying system from the crucial threats that may cause phishing attacks.

Threat modelling is an approach that helps security analysts to classify the risks in the initial phase of the development process of an application. It also helps in the adoption of viable security measures against the identified threats, which will help to mitigate them. Hence, in this paper, we adopt the threat-modelling approach to identify and mitigate phishing attacks in IoT devices by analyzing the potential vulnerabilities, threats, and weak security measures that were implemented. By employing threat modelling, we identify the flaws that remained in IoT devices during the design phase. To better illustrate the proof-of-concept, we considered two smart-use cases, i.e., a smart autonomous vehicular system (AVS) and smart home system that best suit the applications and services of IoT in daily life. The key contributions of this work are as follows:

- We addressed two significant IoT use cases, i.e., smart AVS and smart home use cases, to identify the potential cyber-threats and vulnerabilities in them;
- A STRIDE-based threat-modelling approach is proposed to identify the potential threats and vulnerabilities that can cause phishing attacks in two significant IoT use cases;
- We further proposed mitigation strategies to protect and secure the identified vulnerabilities and threats in the smart AVS and smart home use-cases.

The rest of the paper is arranged as follows. Section 2 describes the existing work done to detect phishing using various approaches. In Section 3, we describe the technical approaches that are widely adopted to perform email phishing. Section 4 discusses the smart use cases considered for this work, in detail. After this, we describe the proposed methodology for identifying the phishing attacks in IoT use cases using the threat modelling approach, in Section 5. Section 6 presents the results along with discussion. Finally, we conclude the whole paper in Section 7.

## 2. Related Work

Phishing attacks comprise emails, messages, etc., containing malicious content or attempting to persuade a user to click on a link and unknowingly share his/her personal information and/or credentials with the attacker. To date, many techniques and strategies have been proposed to detect and stop phishing attacks. Xue et al. [26] proposed a method to identify phishing emails using the persuasion principle [27], which increases the accuracy of machine-learning algorithms. Feature selection in an email is carried out using the information gain algorithm. The persuasion principle counts if the specific word for the features is present in the email. The authors used the first three principles of persuasion [27], i.e., reciprocity, scarcity, and authority, out of six principles, for feature selection. The authors used three machine-learning algorithms, including K-Nearest neighbours, decision tree, and Naive Bayes, to detect malicious emails. The final results showed an accuracy of 99.6% with 25 selected features. Hiroki et al. [28] also detected malicious emails using the persuasion [27] method, but with the deep learning technique.

Gunikhan et al. [29] identified phishing emails using binary search feature selection. To rank the features, a Pearson correlation coefficient algorithm was also used. The features contain the body of the email, the subject of the email, hyperlinks in the email, and the readability of the email content. The overall algorithm contains a feature-ranking algorithm and a feature-selection algorithm to rank and find the best features that can be used to improve the accuracy.

Ozgur et al. [30] worked on phishing email detection from URLs using machine learning. The authors used various classification algorithms, and the features were used based on natural language processing (NLP). The dataset was refined by some data pre-processing techniques, a word-decomposer module, a random word-detection module, and a malicious analysis module. Similarly, Yong et al. [31] worked on the detection of phishing emails using an improved recurrent convolutional neural network (RCNN) approach, in which the RCNN model is improved using bi-directional long short-term memory (LSTM). The model is developed with a multilevel vector and attention strategy that simultaneously classifies the emails based on header, body, level of character, and level of word of the email. When compared with LSTM and CNN, the proposed model had promising results, with 99.84% accuracy. Likewise, Rabab et al. [32] developed a tool to detect the source code of the phishing site that is sent as an attachment with an email. A decision-tree machine-learning algorithm is used as a detection algorithm and suggests anti-phishing strategies. The tools work by allowing the users to save their messages; then, it checks for phishing links in the messages and shows them in the form of a report.

A few studies have also focused on phishing attack detection in IoT devices. Venkatraman et al. [33] classified spam emails for IoT using the semantic similarity approach. This covers the existing deficiencies in spam detection techniques due to an inefficient detection approach because of the context-sensitive nature of the words. The context-sensitive nature creates word polysemy and ambiguity; the authors solved this issue by using the Naive Bayes classification witha semantic similarity approach. The module works in two steps: the first step is to generate the spam and non-spam emails and the second step is to automatically detect them. Likewise, Gupta et al. [34] focused on phishing attacks and their emergence in IoT devices. The authors discussed the existing literature regarding the tools and techniques used for phishing attacks. For example, phishing can be performed using compromised servers and websites, through a botnet, port redirection, social engineering attacks (including website phishing, email phishing, and spear-phishing), malware-based phishing, etc. Various approaches are discussed for use in phishing detection, for instance, phishing detection using features (i.e., email-body-based features, subject-based features, URL-based features, script-based features, and sender/receiver-based features). Other techniques include user awareness, education regarding phishing and the adoption of software-based defence techniques such as network-level protection and client/server-side tools. Similarly, Wenjuan et al. [35] used semi-supervised learning to classify spam emails. This approach is enhanced using multi-view, difference-based classification, which provides abundant information for classification. They name the model as a disagreement-based model that is an ensemble of different classifiers trained in different views. Disagreement-based learning works through the collaboration of different learners, which exploit the unlabeled examples to maintain the large difference in opinion between the base learners. Experimentation with different datasets showed that the proposed approach outperforms the other algorithms.

The above works show the techniques which were adopted to identify the phishing/email phishing for different contexts by adopting various strategies such as the persuasion principle [27], bio-inspired ensemble parallel enhancement algorithm, semantic similarity approach, binary search feature selection, and use of different machine learning techniques, etc. The approaches discussed here can detect phishing but are unable to identify the reasons for and threats that could lead to phishing attacks. Therefore, in this paper, we adopt a threat-modelling approach to identify and mitigate threats in the design phase of the system, before it is physically deployed. Unlike the existing approaches, the proposed threat-modelling approach will highlight the loopholes in each IoT device (of an underlying use case) and other aspects that may allow an attacker to perform phishing attacks. Moreover, the proposed mitigation strategies can assist the IoT vendors and security researchers in considering these remedies and reducing the attack surface during the design phase of an IoT system, to prevent the IoT devices from phishing attacks.

## 3. Technical Approaches of Phishing

There has been a growing pattern of deploying new phishing attacks via digital technology including smartphones and social media. The widespread use of social media provides a fertile base for phishing attacks [36]. According to [11], phishing is comprised of three main components, which include: medium of phishing, vector of phishing, and technical approaches of phishing. The medium of phishing is the source of the attack, such as the internet, text messages, and voice messages. The vector of phishing is the medium through which the phishing attack is carried out, such as emails, websites, and instant messages. The technical approaches are the means by which successful phishing attacks are performed through the adoption of some social engineering techniques. The phisher tricks the victim by adopting these technical approaches; hence, it is mandatory for threat analysts to acquainted with these approaches to carry out the imminent threat modelling. There are many technical approaches to phishing; however, the most commonly adopted technical approaches are listed and described in the following subsections.

### 3.1. Spear Phishing

Spear phishing is a technique of sending spam emails containing malware concealed inside embedded links and attachments that seem to come from reputable sources (e.g., a trustworthy and well-known corporation) [37]. The most prevalent form of phishing attack used by hackers at present is spear phishing. Spear-phishing attacks are more successful than conventional email attacks as they utilize specially designed emails that mimic a source known to the victim. The contents of the email are important to the victim, and do not allow the victim to suspect anything [11]. This means that there is a high chance that the victim succubs to the attack. In 2018, Kaspersky found almost 1000 spear phishing assaults, including 83 distinct attacks targeting American-based educational institutions and 21 other UK universities that were attacked [36]. The intention of attackers in spear-phishing is also to perform an advanced persistent threat (APT), as it is based on a targeted assault and the phisher can launch the assault on a single person, community or entity.

### 3.2. Tabnabbing

Tabnabbing [38] is another type of phishing attack, first introduced by an inventive lead of Firefox named Aza Raskin in the year 2010 [39]. Tabnabbing can be easily understood by describing it as “Tab Kidnapping” in a web browser. The phisher first sends a link to a phishing email to the victim, which opens a malicious website in another tab once clicked. This tab changes its favicon and tab title to look like a legitimate website, such as Facebook, Gmail, LinkedIn, etc. When the user accesses that tab and monitors the phishing login page, he/she logs in thinking that the login session has expired. This type of attack is more successful when the user has opened several tabs and forgets the contents of each specific tab. According to Panda Labs, this new type of phishing attack is on the rise. It mostly targets popular web browsers, such as Internet Explorer and Firefox, opening several tabs with fake material. The attack is performed using a social engineering toolkit created by the lab. The toolkit contains various attack methods, and one of them is tabnabbing by initializing the website attack vector. This attack vector allows the phisher to generate a clone of the legitimate website. The link is then sent to the victim through an email. This will show the clone page to the victim and if, they enter their username and password to log in to clone site, mimicing a popular site such as Gmail, Facebook, and Twitter, their information is received by the phisher.

### 3.3. Whaling

In the context of a targeted attack, Whaling is equivalent to spear-phishing, except the target comprises of high-level employees who have a higher level of access to knowledge or services within a company [40]. To form a more aggressive attack, the phisher, in this type of attack, spends more time sending fraudulent contents via email to convince the victim to click on a link or download attachments. If this activity is carried out, a backdoor or keylogger is installed on the victim’s machine, which sends sensitive information to the attacker. Whaling is performed on high-level targets such as high-ranked government employees or business organizers; attacks on these entities were recorded in 2008, 2010, and 2011. In 2008, several US CEOs received a subpoena with a malicious attachment that installed malware upon viewing. Other successful whaling attack victims include the Australian Prime Minister’s office, Epsilon mailing list service, the Canadian government, RSA SecurID, Oak Ridge National Laboratory, and HBGary Federal in 2010 and 2011 [41].

### 3.4. Phishing Kits

Another way to perform phishing attacks is through phishing kits. Phishing kits allow the attackers to generate malicious websites, emails, and scripts that require no advanced level of programming skills [42]. Phishing kits do not play a vital role in phishing victims’ data, but do assist in the implementation of phishing scams. Phishing kits are available for free or through proper payment to cyber-criminals. However, free kits are not suitable for use, as they steal the personal information of the user and send them back to the phisher. There is also a competence among kit creators that relies on the trustworthiness, availability, ease of use, and security concerns of their kits. Phishing kits can, therefore, be used to send malicious emails or create a phishing website to deceive the victim [43]. Several attacks were reported by [44] which use phishing kits. For example, 10% of the websites active in 2013 reported phishing kits attacks, 120–160 criminal activities were detected for SMTP-based email phishing kit operators, etc. [43].

### 3.5. Drive-by-Download

This is a technique of unintentionally running a virus or a malicious shellcode by visiting a malicious website or responding to an email. As described by [11], this can also be carried out by maliciously using a JavaScript code to exploit the vulnerabilities in a browser hosted using a server, or they can be injected into a website via email. Then, a malicious web page is opened containing a malicious JavaScript code that exploits the web browser vulnerabilities. In a successful exploit, the malware is downloaded on the system, which, as a result, becomes part of the botnet [45]. Drive-by-download has become a very effective attack, for several reasons. For example, there are large-scale web client flaws, composed of 15% common vulnerabilities and exposures (CVE), found in repository reports [46]. Moreover, 45% of internet users used outdated browsers with security problems [47].

### 3.6. Social Engineering

Social engineering is one of the most devastating phishing attack methods, which involves phishers playing into the confidence of the victim, feelings such as compassion or anxiety, the desire to assist, and ignorance of how to accomplish their goal. Social engineering is focused on diverting the victim from making reasonable decisions, causing the victim to make irrational decisions. Fear, envy, intrigue, rage, friendship, loyalty, ambition, selflessness, a sense of obligation and supremacy are examples of such emotions [11]. By taking advantage of these emotions, the attacker causes the victim to reveal his/her personal information and assets. Such actions derive from the defensive predisposition leading one to take precautionary, urgent action. This type of phishing attack is performed using a website, email, voice or text messages, social media, etc. People usually believe that social engineering is easy to detect, but researchers have stated that they respond poorly when detecting deception and lies [48]. Kevin Mitnick’s notorious attacks demonstrated how damaging advanced attacks using social engineering can be for information protection efforts in both corporations and public agencies [49]. This implies that the social engineering technique can be very effective in persuading victims to perform notorious activities.

## 4. Smart Use Cases

In this section, we present two smart-use cases, i.e., smart AVS and smart home, that we considered in this study, due to their increasing trend and enormous importance in our daily life. Both use-cases are IoT-enabled and can be controlled via the internet. The smart AVS is the most astonishing innovation of this age due to its automated driving and controlling functionalities. There are numerous applications of smart AVS; for instance, they are used in airplanes (auto-pilot mode), submarines, cargo vehicles, and smart cars. In this work, we focused on the application of smart AVS in smart cars. A detailed description of smart AVS is provided in Section 4.1. Similarly, the smart home use-case contains different IoT devices, which can be controlled by a user through an Android application via the internet. A detailed description of a smart home system is given in Section 4.2.

### 4.1. Smart Autonomous Vehicular System (AVS) Use Case

Smart AVS is designed to automate the process of driving to reduce the risk of accidents and make driving decisions intelligently. In this work, we focus on the application of smart AVS in smart cars. Based on the functionalities of smart AVS incorporation, a smart car is categorized into five levels (Level-1 to Level-5), as defined by the society of automotive engineers (SAE) [50]. These levels are briefly described in Table 1.

The Level-1 cars are provided with different electronic control units (ECUs), which require the driver’s inclusion to fully control the vehicle. Many vehicles are operating on Level-1, such as the 2018 Toyota Corolla and 2018 Nissan Sentra [51]. In Level-2 cars, the smart AVS can take control of some functions of the car, e.g., steering movement with lane keeping, but the driver remains responsible. Examples of Level-2 autonomous vehicles include Volvo pilot assist, Tesla autopilot, and Audi traffic jam assists [51]. Level-3 cars are the inception of smart cars without drivers. These vehicles can drive themselves under some specific conditions [52]. BMW [53] and Audi 8 [51] are Level-3 autonomous cars available on the market. Level-4 cars are fully automated, and can be driven independently of the driver. Recently, Waymo developed a Level-4 autonomous car with a mobile application to drive passengers without drivers [54]. The Level-5 cars are fully automated, with no driver controls. At present, there are no Level-5 autonomous cars on the market, but they are expected to arrive by the year 2024 in very low volumes [55].

**Figure 2:**
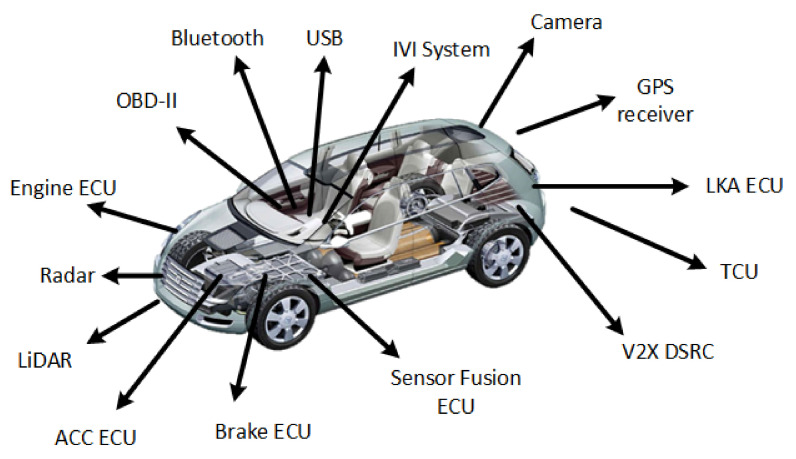
Smart Autonomous Vehicular System (AVS).

To proceed with the proposed threat-modelling approach to phishing attack identification, we considered the example of a highly automated BMW Level-3 smart AVS [53], which comprises different sensors and actuators, such as a camera, radar, LiDAR and ECUs, as illustrated in Figure 2. For a better understanding of the threat-modelling approach, we divided the smart AVS into three zones, i.e., the sensor zone, cloud zone, and consumer zone. The sensor zone contains the sensors and actuators, such as ECUs, camera, and radar. On the other hand, the cloud zone contains the components that are accessed wirelessly, such as GPS receiver, gateway, telematics control unit (TCU), vehicle to everything (V2X), and dedicated short-range communications (DSRC). Similarly, the consumer zone contains the user/driver that controls the vehicle, either manually or using a smart AVS.

#### 4.1.1. Sensor Zone

In the sensor zone, there are various types of sensors and actuators, which are connected with each other to provide the autonomous driving functionality. This zone contains various types of ECUs, which include engine ECU, brake ECU, vehicle ECU, lane-keeping assist (LKA) ECU, adaptive cruise control (ACC) ECU, and sensor fusion ECU, and which are responsible for controlling the engine functions, general vehicle controls, brakes, lane and collision monitoring, etc. All the data within these ECUs flow through the controller area network (CAN). The CAN protocol enables consistency in the data flow and control decisions of different ECUs through CAN packet frames. The CAN packet frames consist of many fields, such as data, data Id, data length code (DLC), and cyclic redundancy check (CRC). Due to its message-oriented nature, the CAN does not operate on the transmitter/receiver address; instead, it contains a unique ID for each value. For example, a CAN frame with Id=0x10 may have the value of engine ECU, and a frame with the Id=0x50 may have the value of LKA ECU [56].

The other sensors that are used to control the vehicle include cameras, radar, and LiDAR. The cameras collect the images of all objects on the road after interpreting the objects. The cameras are placed on top of the roof, which covers a 360 degree view, ranging up to 500 m in size. Similarly, LiDAR forms a 3D picture of the objects surrounding the vehicle within 300 m. The LiDARs are placed on the front and rear bumper of the vehicle, as well as on the upper side of the front wheels. Likewise, the radar is used to calculate the speed or velocity of the other objects/vehicles, and accordingly control the speed of the vehicle.

#### 4.1.2. Cloud Zone

The cloud zone contains a gateway, TCU, GPS receiver, and V2X DSRC. In the cloud zone, the vehicle shares the data to a remote control network using a gateway or a global positioning system (GPS). If the vehicle needs to be monitored or tracked, a TCU is used for this purpose. The TCU reveals the state of the vehicle (engine start, engine stop, vehicle moving, etc.) along with its location. In our use case, the TCU helps GPS receiver to communicate with the system and informs the user of the location and state of the vehicle. If the vehicle needs to communicate with some other nearby vehicle, V2X DSRC is utilized for this purpose. The data are shared with other elements, such as vehicles, gateways, traffic lights or signals, and pedestrians, with a high speed and a high frequency.

#### 4.1.3. Consumer Zone

The consumer zone comprises a user interface, on-board diagnostic-II (OBD-II), and an in-vehicular information (IVI) system with optional Bluetooth and a universal serial bus (USB) port. Through the client zone functions, the user can control the vehicle by manual driving, or by switching the autonomous Level-3 for self-driving. To switch to self-driving, the autonomous system interacts with the OBD-II port that is responsible for diagnosing various types of service, e.g., monitoring engine power, distance, speed calculations, etc. Besides this, the IVI system provides information that is particularly inaccessible to drivers, such as road and traffic situation, map navigation, weather forecast, hazards and notification of obstacles. All this information is expressed to the user through emails, short message service (SMS) or voice mails. Thus, the users can easily understand the road environments and visual perception. Moreover, the IVI system can warn drivers of technological vehicle defects and risky tunnel incidents. Therefore, they are able to increase security and safety.

In order to identify the potential threats that may lead to a phishing attack, we will analyze all the components, defined in three zones of the smart car use-case through the proposed threat-modelling approach.

### 4.2. Smart Home Use Case

The smart home use-case is considered as a generic use-case. In our smart home use-case, the IoT devices are controlled via the Azure server through the IoT field gateway. To better understand this, we divided the smart home use-case into five zones, i.e., IoT device zone, IoT field gateway zone, IoT cloud gateway zone, Azure zone, and consumer zone.

**Figure 3:**
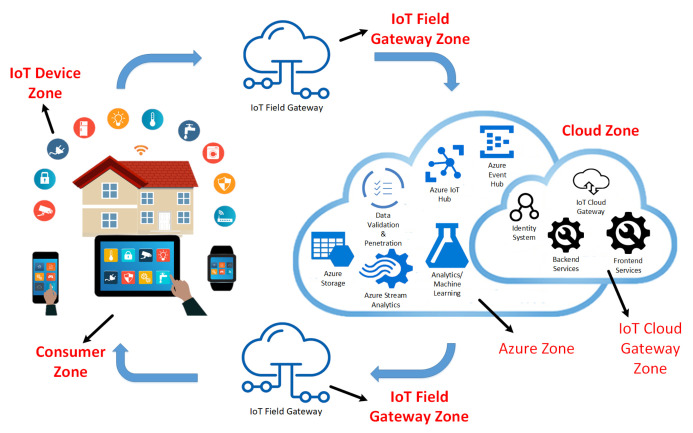
Smart Home System.

The IoT device zone contains all the IoT sensors and actuators, which obtain different sensing values from the environment and are operated using the actuators. However, the IoT filed gateway zone is directly connected to the IoT end-devices, and it operates by controlling the data flow to or from the devices. Similarly, the Azure zone contains different Azure components that analyze and control all the data coming from IoT devices. Likewise, the IoT cloud gateway zone is responsible for the consumer zone’s communication with the IoT device zone, and contains some front-end and back-end services to manage the cloud gateway. Finally, the consumer zone is simply a client zone in which the user can control or see the status of IoT devices using a smartphone or tablet after installing an application that shows the interface of IoT devices. The smart home use-case considered for this work is presented in Figure 3. All the zones are briefly described in the following subsections.

#### 4.2.1. IoT Device Zone

This is the local zone that contains all the physical devices installed in the physical space, such as the personal home of the user. This zone contains a wireless short-range infrastructure, wherein devices can communicate using IoT field gateways and cloud gateways with the Azure zone. The devices could be sensors (sensor, proximity and motion sensor, temperature sensor, sound sensor, moisture sensor, gas sensor, etc.), or actuators (water pump, electric fan, electronic alarm, etc). Other devices could be embedded, such as smart cameras, smart TV, or smartwatch. Based on the type of IoT device, any Linux-based operating system, such as Contiki, RIOT and TinyOS, or a Windows-based OS such as Windows 10 Core IoT OS, could control the operations and services of IoT devices.

#### 4.2.2. IoT Field Gateway Zone

The field gateway zone is comprised of a network device or any other general-purpose computer software system that acts as a communication enabler. Thus, it provides a data transmission medium for devices, and systems for access controls. This zone is directly connected to the end IoT devices and is responsible for communication between the IoT device zone and the Azure zone. The IoT field gateway zone may be unresistant to physical cyber-attacks and has minimal stability and resilience due to its location and limitations regarding technical compatibility.

#### 4.2.3. IoT Cloud Gateway Zone

This zone provides a remote communication medium to the IoT devices, so that they can remotely communicate with the Azure zone through the IoT field gateway. It is also responsible for ensuring that the Azure server is accessible to the consumer and IoT device zone. A user in the consumer zone can remotely access the Azure server from anywhere through the public area network. To isolate all other network traffic from the cloud gateway, as well as from all of its connected devices or field gateways, a cloud gateway may potentially be installed on a network virtualization interface. Moreover, this zone consists of some front-end and back-end services that contain the device information and present it to the client.

#### 4.2.4. Azure Zone

This Azure zone consists of Microsoft Azure server and Azure components such as Azure stream analytics, Azure IoT Hub, and Azure storage. Azure stream analytics is an engine that enables simulations of real-time analytics on data streams of various sources, such as the internet, sensors, actuators, and devices. The Azure event hub is a component of the sensors’ data collection with a very high level of throughput from synchronous sources, while the Azure IoT hub maintains the connection of IoT devices. This zone collects the input data from the IoT cloud gateway zone and passes the information needed for processing to the Azure stream analytics via the Azure IoT hub. The data are stored as recorded information in the Azure storage and can be accessed for further processing using machine-learning analytics.

#### 4.2.5. Consumer Zone

This is the client zone from which the user can control or analyze the IoT devices. This zone follows a command and control mechanism, in which the user sends various requests to the IoT devices over the cloud. The command and control mechanism is performed using an Android application that is connected to the cloud zone with an Azure server. The user can control the IoT devices, such as switching IoT devices such as smart TV, electronic fan, and door on/off, or perform other operations through the Android application.

All the components defined in the five zones of the smart home use-case will be analyzed through the proposed threat-modelling approach to identify the potential threats.

## 5. Proposed Methodology

**Figure 4:**
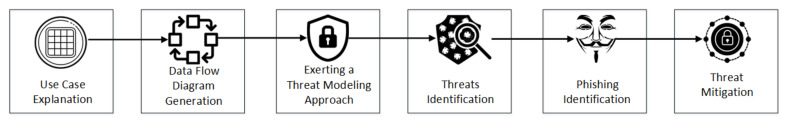
Proposed Methodology for Identification and Mitigation of Phishing Attacks in Internet of Things Smart Home and Smart Autonomous Vehicle System (AVS).

In this section, we apply the proposed threat-modelling approach to identify and mitigate the potential threats that may cause a phishing attack in two smart-use cases. The proposed methodology consists of six major stages, as shown in Figure 4. These stages include use-case reconnaissance, data-flow-diagram generation, exerting a threat-modelling approach, threat identification, phishing threat identification, and threat mitigation. All these stages are described in the following subsections.

### 5.1. Use Case Reconnaissance

Use-case reconnaissance is a preliminary step in the proposed threat modelling approach, in which we gather information on all the stakeholders in an underlying use-case. The stakeholders of a use-case include all IoT devices, sensors, actuators, servers, and network devices deployed or connected in the use-case. Moreover, all the services, device zones, device operations, routing area, and user controls are also the stakeholders of the underlying use-case.

As mentioned earlier in this work, we considered two smart use-cases to demonstrate the proof-of-concept of the proposed threat-modelling approach, used to detect the potential threats in a use-case that may cause phishing attacks. The two smart use-cases considered in this work include smart AVS and smart home. These two use-cases were selected due to their trend of increase and enormous importance in our daily life. In the smart AVS, we followed the architecture of a BMW Level-3 smart AVS [53], which allows for autonomous driving in certain conditions. The driver can use a smartphone application to control the autonomous vehicle. The mobile application shows various pieces of information about the vehicle, such as location, speed, and firmware updates status. The vehicle contains different types of sensor, such as a camera, radar, and LiDAR, and actuators such as ECUs that control it in autonomous driving mode by analyzing the outer environment. The vehicle is also able to communicate with other vehicles using the vehicle-to-everything sensor or a GPS receiver. Based on the way the different components work, we divide the whole use-case into three zones, i.e., sensor zone, cloud zone, and consumer zone. To proceed with the use-case reconnaissance step, we first collected a detailed description of all the stakeholders in the smart AVS use-case, as described in Section 4.1.

A similar step is performed to gather all the stakeholders’ information in the smart home use-case. In our smart home use-case, we considered the IoT devices that can communicate with each other on a local area network and share the data outside the local network over the cloud. The shared data are stored over the cloud, to make it accessible to the user anywhere and at any time. The user uses a smart home mobile application to see the status of devices at home. Based on the IoT devices’ connectivity, online data-sharing and -processing, and consumer usage, we divided the whole system into five zones. These zones are the IoT device zone, IoT field gateway zone, IoT cloud gateway zone, Azure zone, and consumer zone. A detailed description of all the stakeholders in the smart home use-case is given in Section 4.2.

### 5.2. Data Flow Diagram Generation

**Figure 5:**
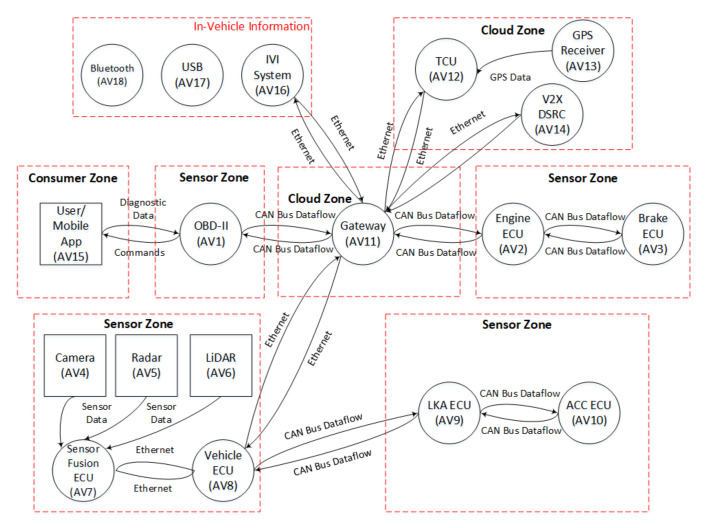
Data Flow Diagram (DFD) of Smart Autonomous Vehicular System (AVS).

**Figure 6:**
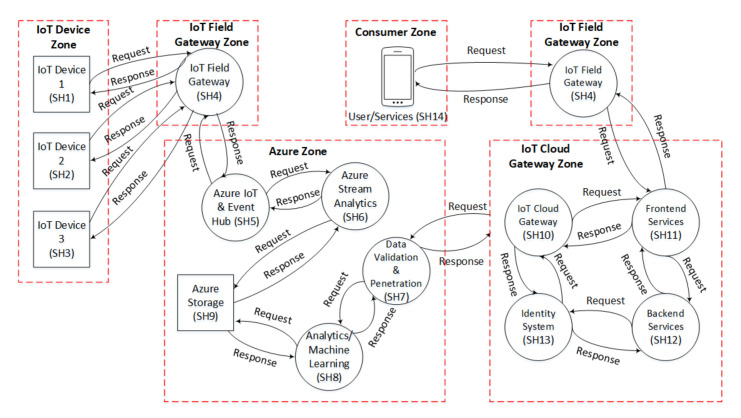
Data Flow Diagram (DFD) of Smart Home System.

After the use-case reconnaissance, the next step is to generate the data flow diagram (DFD) of the use-case, as illustrated in Figure 4. Based on the information gathered in use-case reconnaissance, a DFD is built by connecting each component of a use case. The DFD provides the design overview of a system, to which we apply threat modelling techniques to identify the potential threats in an underlying use-case. To apply a threat-modelling technique on a use-case DFD, various tools such as SecuriCad [57], ThreatModeler [58], and the Microsoft threat modelling (MTM) tool [59] can be used. These tools provide the functionality needed to design a DFD of a use-case and apply the threat-modelling technique to identify the potential threats. We used the MTM [59] tool, as it supports the drawing of the DFD of IoT use-cases such as smart AVS and smart home system. It also classifies the types of threat using the STRIDE threat-modelling approach. Therefore, we use the MTM tool [59] to draw the DFD of both the smart use-cases. The DFDs of both use-cases are displayed in Figure 5 and Figure 6, respectively. The following subsection discusses these DFDs in detail. All the components are represented with their relevant IDs in Table 2. Also, Table 2 presents the zone-wise Ids for each component for both use-cases.

#### 5.2.1. DFD of the Smart AVS Use Case

As discussed earlier, we used the MTM tool [59] to draw the DFD of smart AVS use-cases based on the information gathered in use-case reconnaissance. The MTM [59] tool contains different components in its stencils pane that can be dragged and dropped in the tool’s workplace to draw the DFD of smart AVS. Figure 5 displays the DFD of the smart AVS, which contains different components such as ECUs, cameras, radar, LiDAR, and GPS receiver, as discussed in Section 4. The square boxes with solid black lines in Figure 5, represent the user/mobile application, camera, radar, and LiDAR, while the circles with solid black lines show the ECUs and other components in the system, such as TCU, GPS receiver, OBD-II port, IVI system, and gateway. The IVI system is placed in an in-vehicle interface boundary, with Bluetooth and a USB port for data connection and storage operations. The data flow among the components is shown with black arrows such as Ethernet and CAN bus data flow. The red dotted lines represent each zone, with the corresponding components placed in that zone. The data flow among different components is shown with black arrows.

#### 5.2.2. DFD of Smart Home Use Case

The DFD of the smart home use-case comprises five zones, as shown in Figure 6. These zones include the IoT device zone, IoT field gateway zone, Azure zone, IoT cloud gateway zone, and the consumer zone. Descriptions of all the zones have been given in Section 4.2. The square boxes with solid black lines in the diagram represent the IoT devices and Azure storage. The black circles show the processing components, such as the IoT field gateway, IoT cloud gateway, and Azure stream analytics. Each zone is separated by the red dotted boundary lines. The data flow among the components and zones is represented with black arrows.

In the DFDs of both use-cases, we assigned a short identity (Id) to each component to easily analyze the behaviour of identified threats. For example, the gateway is represented by Id “AV11” in Figure 5. Similarly, the IoT field gateway is represented by Id “SH4”. The Ids of smart AVS components are represented by the prefix “AV”, while the Ids of smart home components are represented by the prefix “SH”.

### 5.3. Exerting a Threat Modelling Approach

Once the DFD of an underlying use case is designed, the next step is to exert a threat-modelling approach. There are various threat modelling approaches such as VAST, PASTA, LINDDUN, CVSS, OCTAVE, and STRIDE, and detailed descriptions of these approaches are given in [60]. In our case, the MTM tool [59] supports the STRIDE threat-modelling approach; therefore, we applied STRIDE to proceed with the threat-modelling of both smart use-cases.

STRIDE is a combination of different threats, such as spoofing, tampering, repudiation, information disclosure, denial of service (DoS), and elevation of privilege. Although the STRIDE approach is time-consuming, it individually analyzes each element present in its DFD [61]. The threats identified by STRIDE also depict violations of basic security requirements. Table 3 presents a mapping of each STRIDE threat with the corresponding security measures.

### 5.4. Threat Identification

After exerting the threat-modelling approach, the next step is threat identification, as displayed in Figure 4. As we applied the STRIDE threat-modelling technique in MTM [59] tool, it generated a threat report of each component of a given DFD. Afterwards, we listed all the threats separately in the results Section 6. The listed threats show how the components can be compromised by a specific threat. We also described the assets which were affected by each STRIDE threat, and their mapping, with security requirement violations.

### 5.5. Phishing Threats Identification

Once we listed all the threats identified using the STRIDE technique, we then analyzed which of the identified threats may cause a phishing attack. Phishing attacks pose a precarious threat to all users of the Internet and are hard to locate or protect against, as it they not explicitly malicious. As discussed earlier regarding in phishing attacks, the attacker applies different techniques to obtain the device/user credentials, and then use these for malicious purposes. In the phishing threats identification stage, we discovered which of the identified threats may cause information leakages, which ultimately lead to phishing attacks. Based on the information leakage threats, we performed zone-wise analysis to identify how an attacker can exploit these threats to perform a phishing attack and gain sensitive information. A detailed analysis of phishing threats for both use-cases is given in Section 6.3 and Section 6.4.

### 5.6. Threat Mitigation Techniques

After identifying the potential threats in both use cases, we proposed threat mitigation techniques to protect the underlying use-case from the cyber-attacks. To propose these threat mitigation techniques, we reviewed some existing research works, i.e., [1,62,63,64] for IoT threat mitigation and selected the best possible remedies to minimize the threats in both smart-use cases. The proposed threat mitigation techniques for both use-cases are described in Section 6.5 and Section 6.6.

## 6. Results and Discussion

In this section, we first discuss the potential threats identified in both use-cases, using the STRIDE threat-modelling approach. To summarize the identified threats, we show the threats to each component in a table, and the total number of occurrences of each threat in a bar graph. After this, we identify the threats that would be responsible for performing phishing attacks in both use-cases. Finally, we propose threat mitigation techniques to stop phishing and other threats for both use-cases.

### 6.1. Threat Identification in Smart AVS Use Case

**i. Spoofing Threats:** In the smart AVS use-case, we found a total of 12 spoofing threats. Most of the reported threats were found in the sensor zone. If an attacker attempts to send a spoofed CAN message in the sensor zone, it can disable the ECUs and OBD-II. As a result, the vehicle-monitoring system may fail and cause a severe incident. Similarly, the gateway and GPS receiver could be spoofed by the adversary to deliver malignant data to the users in connected vehicular systems due to the lack of data validation mechanisms in the GPS. Likewise, the spoofing of a camera, radar, and LiDAR sensors attached to the vehicles can lead the sensor fusion ECU to malfunction during automated driving.

**ii. Tampering Threats:** In this category, we found a total of six threats in the smart AVS use-case. Among these threats, four were found in the sensor zone, while two threats were found in the cloud zone, as mentioned in Table 4. If an attacker exploits these threats, then this can tamper the data transmission among vehicle ECU, engine ECU, brake ECU, and sensor fusion ECU, which will ultimately break down automated or even manual driving. Likewise, if an adversary attempts to tamper with the data transmission to TCU and V2X DSRC, this can result in the sending of false information to all the connected vehicles through GPS or wireless, which may cause a catastrophic incident.

**iii. Repudiation Threats:** With the STRIDE threat-modelling approach, we found a total of four repudiation threats that could allow an attacker to gain illicit access to the smart AVS and malfunction the automated driving. These threats were found in the cloud zone and consumer zone, as displayed in Table 4. By exploiting these security threats, an intruder can misinform both the smart AVS and the driver by giving the wrong traffic and environment information.

**iv. Information Disclosure Threats:** In this category, we found a total of 13 threats that may cause information disclosure. Most of the threats were found in the sensor zone and cloud zone, and were mainly found in ECUs, TCU, OBD-II, and V2X DSRC, as shown in Table 4 By exploiting these threats, an adversary can compromise the confidentiality and privacy of the user.

**v. Denial of Service (DoS) Threats:** In this category, a total of 13 threats were reported that may cause a DoS attack, as mentioned in Table 4. The reported DoS threats include almost all the entities in the use-case, which mainly includethe gateway, OBD-II, ECUs, IVI system, TCU, V2X DSRC, GPS receiver, and mobile applications. If an attacker instigates a DoS attack on these entities, it may halt the smart AVS and also affect manual driving.

**vi. Elevation of Privilege Threats:** This threat exploits authorization, and the adversary illegally gains the privileges of all resources without the permission of the user. In this category, we found a total of 12 threats in all three zones, as displayed in Table 4. By exploiting these threats, an adversary can take over the whole smart AVS, which may cause a drastic incident, such as jamming the whole smart AVS during autonomous driving mode. Furthermore, the adversary can install malware, spyware, etc., in order to extract sensitive information, such as login credentials, vehicle location, and movements.

### 6.2. Threat Identification in Smart Home Use Case

**i. Spoofing Threats:** In the smart home use-case, we found a total of 10 reported spoofing threats. Most of the spoofing threats were found in the IoT device zone and Azure zone, as shown in Tabletab:Threat Mapping. These threats include the use of a similar authentication token in IoT devices that can lead to all the IoT devices being hijacked if one’s token is compromised. Due to the lack of auditing, i.e., not limiting access to registered users, the IoT field gateway and IoT cloud gateway could be abused. If these gateways are compromised, the adversary would be able to send malformed IoT device data by breaching them using admin privileges. In the Azure zone, the adversary can exploit the Azure administrator authentication and, hence, access the Azure subscription.

**ii. Tampering Threats:** In this category, a total of nine threats were reported, as displayed in Table 4. Most of them were found in the IoT device zone and IoT cloud gateway zone. The IoT device zone-tampering threats may lead to the manipulation of identified unpatched device flaws, the theft of security key components, the initiation of offline assaults by modifying device operating systems, and the interception of encrypted traffic sent to devices. In this way, an attacker can obtain and modify critical system information, such as sensor and actuator data. HOwever, in the case of IoT field gateway tampering threats, an adversary can obtain confidential information and tamper with the original data by executing a malicious code. Regarding the IoT cloud zone, the adversary can tamper or modify the binaries in back-end services using reverse-engineering tools such as Sandbox and IDA Pro. Similarly, by exploiting the Azure zone tampering threats, an attacker can change the data stored in Azure storage, malfunctioning all entities of a smart home that are decided based on Azure storage data.

**iii. Repudiation Threats:** In this category, we found a total of eight repudiation threats, as shown in Table 4. Most of the repudiation threats were reported in the IoT device zone and Azure zone. By exploiting these threats, an adversary can gain unauthorised access to the smart home system and malfunction the normal working. The repudiation threats in the IoT device zone can allow an attacker to terminate IoT devices’ access to the field gateway and could send fake data from the devices to the user in the consumer zone. Similarly, the threats recorded in the Azure Zone can cause account theft and sensitive data leakage.

**iv. Information Disclosure Threats:** The MTM [59] tool reported a total of 10 threats that can disclose sensitive information to an attacker. Most of these threats were found in the IoT device zone and Azure zone, as displayed in Tabletab:Threat Mapping. These threats can allow an adversary to eavesdrop on communication between the IoT device and the IoT field gateway. Likewise, the threats in the consumer zone can allow an attacker to jailbreak the mobile application and steal sensitive information.

**v. Denial of Service Threats:** In this category, a total of 13 threats were reported that can lead to denial of service. Tabletab:Threat Mapping depicts that these threats were recorded in almost all zones of the smart home use-case. By sending a flood of malformed packets, an attacker can limit the availability of services and resources. Moreover, the attacker can also block access by the Android application or application programming interface (API) through DoS.

**vi. Elevation of Privilege Threats:** In this category, we found a total of 12 threats, as shown in Tabletab:Threat Mapping. Most of the threats were reported in the IoT device zone, Azure zone and IoT cloud gateway zone. These threats arose due to open or unused services in IoT devices, utilization of device token credentials to gain high privileges, unnecessary access to protected features, and the triggering of unauthorised commands. By exploiting these threats, an adversary can gain complete control over the devices, and perform malicious activities to harm smart home users.

**Figure 7:**
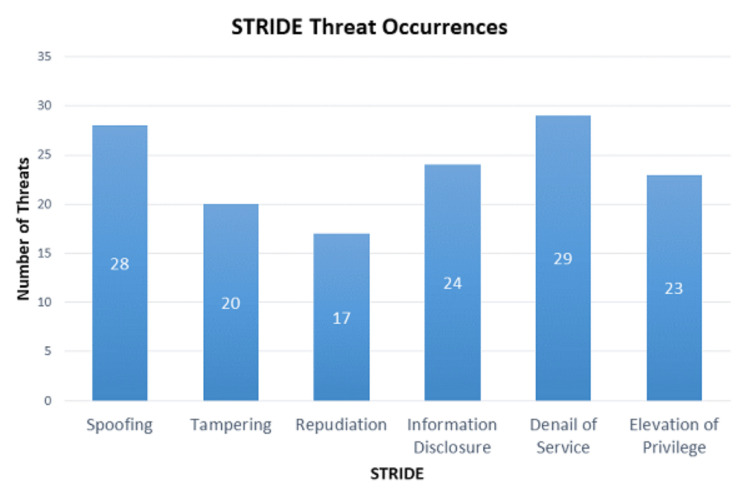
STRIDE Threats Summary.

The STRIDE threats in each component of both systems are shown in Table 4. The AV Assets and SH Assets columns in Table 4 represent the components affected by the corresponding STRIDE threat in the smart AVS and smart home system, respectively, as a component Id. The summary of all the STRIDE threats, and the total number of occurrences of each threat in both use-cases, is shown in Figure 7.

### 6.3. Phishing Threats in Smart AVS Use Case

In this section, we analyze all the threats in the smart AVS use-case reported in the threat identification stage to figure out which of the identified threats can lead to phishing attacks. As discussed earlier, in a phishing attack, the primary goal of the attacker is to gather sensitive information by sending an email, eavesdropping, spoofing, etc. After obtaining the sensitive information, the attacker uses it for malicious purposes, such as wrecking the underlying system in order to harm the end-users. Likewise, we showed that the STRIDE threat-modelling approach identifies six types of threats in an underlying use case, which include spoofing, tampering, repudiation, information disclosure, DoS, and elevation of privilege threats. If we carefully interrelate these identified threats with the primary goal of a phishing attack, (i.e., gaining sensitive information), we can easily figure out which of these threats can expose sensitive information to an attacker. Among these threats, the three threats, i.e., spoofing, information disclosure, and elevation of privilege threats, can make it easier for an attacker to gather the sensitive information of an underlying system/use-case. Since we divided the smart AVS use-case into three zones, here, we analyze how the identified threats can allow an attacker to perform a phishing attack.

**i. Phishing in Sensor Zone:** The sensor zone contains the different sensors and actuators, such as ECUs, camera, ultrasonic sensor, radar, and LiDAR. The sensors provide information to the smart AVS, which it uses to navigate the vehicle and actuate the ECUs—the engine ECU, brake ECU, lane-keeping ECU, etc.—in autonomous driving mode. Moreover, the information gained from the sensors provides an understanding of the external environment to the smart AVS during autonomous driving mode. In the threat identification step, the MTM tool [59] identified five types of STRIDE threat as the sensor zone out of which three threats, i.e., spoofing, information disclosure, and elevation of privilege threats, can allow an attacker to perform a phishing attack.

Spoofing any sensor in the sensor zone can help the attacker to gather information on the protocols, message size, message types (encrypted or textual), etc., used in smart AVS. This information can help the attacker to easily exploit the vulnerabilities of an underlying smart AVS for malicious purposes. Consequently, the exploitation of spoofing threats can allow an attacker to spoof the sensor information, which can ultimately break down normal autonomous driving and cause a serious incident. However, when spoofing sensor data, the attacker should either have physical access or be in proximity to the car. In [65], the authors sent physical signal noise of an ultrasonic sensor from the nearby location of a smart car to disrupt the sensor reading. This attack can lead to the failed detection of obstacles, which can cause a serious accident. Similarly, the information disclosure threats in the sensor zone can allow an adversary to obtain information by network-scanning or an eavesdropping attack. Likewise, the elevation of privilege threats can allow an attacker to tamper with the sensor data, which can affect the vehicle’s navigation during autonomous driving mode.

**ii. Phishing in Cloud Zone:** In the cloud zone, all the information is passed outside the network to communicate with other vehicles or remote users. During threat identification, the MTM [59] tool detected all six types of threat in the cloud zone. Most of the threats found were related to spoofing, information disclosure, the elevation of privilege, and the DoS threats category. Among these, the first three threats can allow an attacker to perform a phishing attack on smart AVS. By exploiting the elevation of privilege threats in the cloud zone, an attacker can take full control of the smart AVS, from which he/she can obtain and spoof sensitive information regarding the GPS location and speed of the car, which can cause delays or disruptions in critical vehicle operations, such as breaks and airbags. Moreover, the attacker can break down the other functionalities of the car to harm the car users.

**iii. Phishing in Consumer Zone:** In the consumer zone, the user/driver physically interacts with the vehicle or uses the autonomous system to drive. The consumer zone is the most lucrative target for adversaries, as it contains an IVI system, Bluetooth, USB, and media player and is also directly connected with the internet access point. In the threat identification stage, we found four types of threat in the consumer zone, which include repudiation, information disclosure, DoS, and elevation of privilege. In the consumer zone, the chances of a phishing attack are higher compared to other zones. The reason for this is that, in this zone, an adversary can send a phishing email to install updates or download some file onto the user’s mobile or the car’s media player, through which an attacker can install malware, spyware, etc. If the user’s mobile is infected with phishing emails, the malware can be transferred to the vehicle, infecting its sensors and actuators. Similarly, if the media player becomes infected with malware or spyware, etc., then the attacker can steal the user’s sensitive information or take full control of the vehicle.

### 6.4. Phishing in Smart Home Use Case

As discussed earlier, the smart home use-case considered in this study is a generic use-case, in which a user can increase or decrease the number of IoT devices. The user is also allowed to use Android applications on multiple smartphones to increase the number of users in the consumer zone. In this section, we analyze the threats in a smart home use-case, as reported in the threat identification stage, to highlight which of the identified threats can cause phishing attacks. Since we divided the smart home use-case into five zones, here, we analyze how the identified threats can allow an attacker to perform a phishing attack.

**i. Phishing in IoT Device Zone:** The IoT device zone is connected to an IoT field gateway that is responsible for transferring IoT devices’ data over the cloud. In the threat identification stage, we found all six types of STRIDE threat in the IoT device zone, of which three types of threat, i.e., spoofing, information disclosure, and elevation of privileges, lead to phishing attacks. We noticed the usage of the same authentication token in all the IoT devices; the adversary could spoof all the IoT devices if one is compromised. Therefore, the sensitive information of IoT devices would be disclosed to the adversary. In this way, he/she would be allowed to send fake messages or emails to the user in the consumer zone, i.e., sending false data of the scheduled statistics showing the condition and usage of devices. In addition, by sending an email to the customer zone with a link that launches the adversary’s malignant website, imitating the IoT devices’ actual login page, the adversary may request the user’s login credentials. The user would then submit their private credentials to the adversary. Hence, spoofing and information disclosure are sources of phishing in this zone.

**ii. Phishing in IoT Field Gateway Zone:** We reported spoofing, tampering, repudiation, information disclosure, and denial of service in this zone. There is a lack of auditing in the IoT field gateway; hence, it could be spoofed with false information. The adversary would be able to eavesdrop on the communication between the IoT device zone and IoT cloud zone, and may send false IoT device alerts to the user. The adversary could modify the data flow between devices and the cloud by injecting malicious code into the data. These data, when displayed on the user’s mobile application, would install the injected malicious code on the phone. In this way, all the personal information of the user would be available to the adversary. Hence, spoofing and information disclosure threats can cause phishing attacks in this zone.

**iii. Phishing in Cloud and Azure Zone:** The cloud zone comprises the Azure zone and the IoT cloud gateway zone. The Azure zone performs the data-processing and control operations by performing data validation and penetration testing. The Azure server also performs different analytics and machine-learning operations. In the threat identification section, we reported spoofing, tampering, repudiation, information disclosure, DoS, and elevation of privileges in the cloud zone. These threats would result in an insecure medium of communication between the Azure storage and the consumer, remote code execution, man-in-the-middle attack, account theft, etc., in the Azure server. Due to spoofing, the adversary attempts to obtain unauthorized access to the IoT cloud gateway. By adopting spoofing attempts, modifications to the data are made by the adversary, which can affect the integrity of the data stored in Azure storage. Hence, the user will obtain false information on the status of the devices. Therefore, the adversary could obtain the phished email schedule for the user by spoofing the cloud zone by changing the header and subject of the emails to will look as if they were received by the vendor of the devices. In this way, the adversary can demand personal information, such as credit card number and PIN code, to request the online purchasing of a in the future. All the personal information of the user would be disclosed to the adversary. As well as this, the frontend and backend services could be spoofed in the IoT cloud gateway zone by modifying the strings, OpCodes, and APIs in these services using reverse-engineering tools. Hence, spoofing, information disclosure and the elevation of privileges may cause phishing attacks in this zone.

**iv. Phishing in Consumer Zone:** This is the user zone, which can have single or multiple users, with the Android application installed on their smartphones or tablets. This is the best place for phishers to spoof the user by sending phishing emails or messages. In this zone, we reported repudiation, information disclosure, denial of service, and elevation of privilege threats. As there is no track of what device information is passed to the user through IoT cloud gateway due to lack of auditing, the user has no track of traffic logs in a mobile application. Therefore, the adversary can deny privileged access to the user by interrupting the traffic with malicious data, affecting the repudiation. In this way, all the user’s information is disclosed to the adversary—the traffic flow, mobile application access, etc. The adversary can send malicious emails to the user’s mobile phone. Furthermore, all the threats identified in other zones can influence the consumer zone, because the user is responsible for controlling the smart home system.

### 6.5. Threat Mitigation for Smart AVS Use Case

Threat mitigation strategies allow the users and device vendors to free the system of all the threats identified during threat modelling. Therefore, we propose threat mitigation is implemented for the threats identified during the threat identification stage. As we identified STRIDE threats that could lead to phishing attacks in both use-cases in the previous section, we cover threat mitigation for all the STRIDE threats, which would also stop phishing attacks in both systems.

Spoofing in the CAN protocol can be minimized by implementing data validation and message authentication codes. For example, to alter data in transit, by utilizing direct physical links to the network or indirect interference with the Engine ECU, e.g., altering a V2X packet to add an immediate danger, the car may execute an emergency brake. This will authenticate the data flow from the CAN bus and help to reduce the risk of LKA ECU, as discussed above. Similarly, spoofing in the GPS receiver can be mitigated by combining the GPS receiver with an additional source of data validation and authentication schemes. Likewise, integrating the camera and sensor with a gateway module can help to reduce spoofing in these components.

Similarly, implementation of proper auditing in the gateway would keep track of all the information flowing through it. Additionally, firmware updates are needed for components to work properly and securely. If the connection to the delivery server is not encrypted and is not limited to the specific components that need updates, the update package files could be malignant or accessed by unauthorized users. For example, if only OBD-II firmware needs to be updated, the updates must be downloaded for this component only. If any additional updates are downloaded for other components, there must be a security mechanism to check whether the files are secure and registered from a trusted server.

### 6.6. Threat Mitigation for Smart Home Use Case

The first threat related to spoofing reported in this use-case is the use of a similar authentication token in IoT devices, which can be mitigated by initiating the different shared access signatures (SAS) in each device. In addition, the devices should be authenticated with transport layer security (TLS). For devices that do not accommodate complete asymmetric cryptography, the network should support the use of a pre-shared key (PSK). This can make each device more secure using the different authentication tokens, by limiting access to the controls. The IoT field and cloud gateways must be secured against unauthorized access by enabling proper auditing, using auditing rules in a firewall, according to the security requirements. Moreover, the security can be enhanced by altering the default login credentials in gateways. It is important to ensure that the link requests and authentication status (success or failure) for specific devices are logged and retained on the field gateway when several devices connect to a field gateway. In situations where the IoT hub credentials for specific devices are held by the field gateway, ensure that auditing is carried out when these credentials are obtained. Establish a mechanism to upload the logs regularly for long-term preservation in Azure storage. This process will also secure confidentiality and reduce the information disclosure threat for the data that flow through these components.

Tampering and repudiation in IoT devices, such as exploitation of the vulnerabilities in unpatched devices, can be mitigated by enabling the proper device firmware updates. The security updates can be implemented through the IoT cloud gateway that passes security updates and is patched to IoT devices. While the IoT field gateway could be protected against unauthorized access by the secure extraction and storing of confidential data to protected hardware storage, such as smart card chips or a trusted platform module (TPM). Moreover, the IoT field gateway could be secured data loss by encrypting the OS and enabling additional partitions on its storage. The execution of unknown or malicious code on the IoT field gateway or IoT devices and Azure server could be stopped by using the secure boot mechanism, which restricts the device to only allow the execution of binaries that are signed by a trusted authority. Denying a user’s actions on Azure storage could be ensured by the use of Azure storage auditing. This will enable the logging of Azure storage and store metrics data. The logs will provide important information related to the Azure zone, such as how and when it is accessed and whixh authentication mechanism was used.

DoS threats can be mitigated by limiting access to unused services and open ports. If there are open ports and unused services in the network, the adversary may gain unauthorized access to them and perform DoS attacks. Moreover, this could lead to a man-in-the-middle attack, through which the adversary could access the information shared among the components and alter it accordingly. Additional security can be inaugurated by encrypting the data communication to prevent DoS threats.

## 7. Conclusions

With the widespread adoption of insecure IoT devices, phishing has become a major threat for all Internet users. Phishing is one of the most serious attacks an adversary can launch, involving the sending of malicious emails or messages with malicious links or counterfeit contents. In recent years, the attention paid by attackers to phishing has increased, as it can be performed by exploiting vulnerabilities in the emerging context of IoT devices.

One way to protect the vulnerabilities of IoT devices is to identify and mitigate the threats present in IoT devices before they are deployed. With this aim in mind, in this paper, we proposed a threat-modelling approach to identify and mitigate potential threats in IoT devices during the initial design phase. To better illustrate the proof-of-concept of the proposed threat-modelling approach, we considered two significant IoT use-cases, i.e., smart AVS and smart home. We used different zones to describe the applications of the connected smart devices in daily life. We adopted STRIDE, a threat-modelling approach tha uses all the system’s components details to reveal the threats present in that system. Hence, we began with the use-case reconnaissance to obtain the detailed information of each stakeholder in both use-cases. Based on the information we gathered, we designed a DFD from both use-cases in a threat-modelling tool. After this, we exerted the STRIDE threat-modelling approach on DFDs of both use-cases to identify the potential threats in the underlying IoT devices. Thereafter, we discerned which of the identified threats could allow an adversary to perform a phishing attack. Finally, we proposed threat-mitigation techniques to secure the IoT against potential threats that may lead to phishing attacks in both systems. The proposed threat-modelling approach is a significant step for security analysts, developers, and IoT device vendors in the identification and security of IoT devices’ vulnerabilities during the initial design phase, to protect them from potential threats.

The proposed treat modelling approach only identifies the threats present in the design phase of a system. Based on the identified threats, we proposed threat-mitigation remedies to preotect the IoT systems against phishing attacks. In future, we aim to develop a prototype of these systems and apply the proposed threat mitigation remedies to the validation of the proposed mitigation techniques, by performing real-time cyber-attacks on these systems. Possible extensions to the work may also be offered, including further refinements of the adopted approach, or the inclusion of additional use-cases.

## Figures and Tables

**Table 1:** Society of Automotive Engineers (SAE) Levels of Autonomous Vehicles.

SAE Level	Key Function
Level-1	Driver assistance with electronic control units (ECUs).
Level-2	Partial automation with some advanced features, such as steering control with auto-lane keeping.
Level-3	High automation with some drive-independent controls.
Level-4	Full automation with complete driver-independent controls.
Level-5	Full automation with all driving functions

**Table 2:** Components Representation of Smart Use Cases.

**Smart Autonomous Vehicular System**	
**Assets**	**Ids**
Sensor Zone	
OBD-II, Engine ECU, Brake ECU, Camera, Radar, LiDAR, Sensor Fusion ECU, Vehicle ECU, LKA ECU, ACC ECU	AV1, AV2, AV3, AV4, AV5, AV6, A7, AV8, AV9, AV10
Cloud Zone	
Gateway, TCU, GPS Receiver, V2X DSRC	AV11, AV12, AV13, AV14
Consumer Zone	
User/Laptop, IVI System, USB, Bluetooth	AV15, AV16, AV17, AV18
**Smart Home System**	
**Assets**	**Ids**
IoT Device Zone	
IoT Device 1, IoT Device 2, IoT Device 3	SH1, SH2, SH3
IoT Field Gateway Zone	
IoT Field Gateway	SH4
IoT Cloud Gateway Zone	
IoT Cloud Gateway, Frontend Services, Backend Services, Identity System	SH10, SH11, SH12, SH13
Azure Zone	
Azure IoT & Event Hub, Azure Stream Analytics, Data Validation & Penetration, Analytics/Machine Learning, Azure Storage	SH5, SH6, SH7, SH8, SH9
Consumer Zone	
User/Service	SH14

**Table 3:** STRIDE Mapping with Security Measures.

STRIDE Threat	Descriptions	Security Violation
Spoofing	Misleading a user or system by illegally accessing authentication information	Authentication
Tampering	Maliciously modifying the original information by accessing it without permission	Integrity
Repudiation	Denying the user’s privileged access by performing malicious actions	Non-repudiation
Information Disclosure	Exposing the sensitive information of the user without permission	Confidentiality
Denial of Service	Denying the network or services access to the valid user	Availability
Elevation of Privilege	Gaining the privileged resources without the user’s permission, to compromise the system	Authorization

**Table 4:** STRIDE Threats Mapping in both Use Cases.

STRIDE	Descriptions	AV Assets	SH Assets	CIA
Spoofing	An adversary can exploit the authentication by performing malicious actions	AV1, AV2, AV3, AV4, AV5, AV6, AV7, AV8, AV9, AV10, AV11, AV13	SH1, SH2, SH3, SH4, SH5, SH6, SH7, SH8, SH9, SH10	Authentication
Tampering	An adversary can steal the stored data and change it accordingly	AV2, AV3, AV7, AV8, AV12, AV14	SH1, SH2, SH3, SH4, SH5, SH9, SH11, SH12, SH13	Integrity
Repudiation	An adversary can gain privileged access	AV11, AV16, AV17, AV18	SH1, SH2, SH3, SH4, SH5, SH6, SH9, SH14,	Non-repudiation
Information Disclosure	An adversary can disclose the sensitive information by taking control over the communication medium	AV1, AV2, AV3, AV7, AV8, AV9, AV10, AV11, AV12, AV13, AV14, AV15, AV16	SH1, SH2, SH3, SH4, SH5, SH6, SH7, SH8, SH9, SH14	Confidentiality
Denial of Service	An adversary can deny the authorized users access	AV1, AV2, AV3, AV7, AV8, AV9, AV10, AV11, AV12, AV13, AV14, AV15, AV16	SH1, SH2, SH3, SH4, SH5, SH6, SH7, SH8, SH9, SH10, SH11, SH12, SH14	Availability
Elevation of Privilege	An adversary can avail the privileged resources	AV1, AV2, AV3, AV7, AV8, AV9, AV10, AV11, AV12, AV14, AV15, AV16, AV18	SH1, SH2, SH3, SH5, SH6, SH7, SH8, SH9, SH10, SH11, SH12, SH14	Authorization

## Data Availability

Not applicable.

## References

[1] Vishwakarma R., Jain A.K. (2020). A survey of DDoS attacking techniques and defence mechanisms in the IoT network. Telecommun. Syst..

[2] Yang Y., Wu L., Yin G., Li L., Zhao H. (2017). A survey on security and privacy issues in Internet-of-Things. IEEE Internet Things J..

[3] Hossain E., Khan I., Un-Noor F., Sikander S.S., Sunny M.S.H. (2019). Application of big data and machine learning in smart grid, and associated security concerns: A review. IEEE Access.

[4] Sikder A.K., Petracca G., Aksu H., Jaeger T., Uluagac A.S. (2018). A survey on sensor-based threats to internet-of-things (iot) devices and applications. arXiv.

[5] Firdous S.N., Baig Z., Valli C., Ibrahim A. Modelling and evaluation of malicious attacks against the iot mqtt protocol. Proceedings of the 2017 IEEE International Conference on Internet of Things (iThings) and IEEE Green Computing and Communications (GreenCom) and IEEE Cyber, Physical and Social Computing (CPSCom) and IEEE Smart Data (SmartData).

[6] Sun H., Xu M., Zhao P. (2020). Modeling Malicious Hacking Data Breach Risks. N. Am. Actuar. J..

[7] Basit A., Zafar M., Liu X., Javed A.R., Jalil Z., Kifayat K. (2020). A comprehensive survey of AI-enabled phishing attacks detection techniques. Telecommun. Syst..

[8] Burda P., Chotza T., Allodi L., Zannone N. Testing the effectiveness of tailored phishing techniques in industry and academia: A field experiment. Proceedings of the 15th International Conference on Availability, Reliability and Security.

[9] Verizon’s 2019 DBIR: Phishing Is the Top Threat Action. https://www.proofpoint.com/us/security-awareness/post/verizons-2019-dbir-phishing-top-threat-action.

[10] Nirmal K., Janet B., Kumar R. (2020). Analyzing and eliminating phishing threats in IoT, network and other Web applications using iterative intersection. Peer-to-Peer Netw. Appl..

[11] Chiew K.L., Yong K.S.C., Tan C.L. (2018). A survey of phishing attacks: Their types, vectors and technical approaches. Expert Syst. Appl..

[12] Gupta B.B., Arachchilage N.A., Psannis K.E. (2018). Defending against phishing attacks: Taxonomy of methods, current issues and future directions. Telecommun. Syst..

[13] Jartelius M. (2020). The 2020 Data Breach Investigations Report—A CSO’s perspective. Netw. Secur..

[14] Aleroud A., Zhou L. (2017). Phishing environments, techniques, and countermeasures: A survey. Comput. Secur..

[15] Halevi T., Lewis J., Memon N. A pilot study of cyber security and privacy related behavior and personality traits. Proceedings of the 22nd International Conference on World Wide Web.

[16] Gupta B.B., Tewari A., Cvitić I., Peraković D., Chang X. (2021). Artificial intelligence empowered emails classifier for Internet of Things based systems in industry 4.0. Wirel. Netw..

[17] Sun H., Wang X., Buyya R., Su J. (2017). CloudEyes: Cloud-based malware detection with reversible sketch for resource-constrained internet of things (IoT) devices. Software Pract. Exp..

[18] 2020 Unit 42 IoT Threat Report. https://iotbusinessnews.com/download/white-papers/UNIT42-IoT-Threat-Report.pdf.

[19] Sharma H., Meenakshi E., Bhatia S.K. A comparative analysis and awareness survey of phishing detection tools. Proceedings of the 2017 2nd IEEE International Conference on Recent Trends in Electronics, Information & Communication Technology (RTEICT).

[20] Bhardwaj A., Sapra V., Kumar A., Kumar N., Arthi S. (2020). Why is phishing still successful?. Comput. Fraud Secur..

[21] Wang D., Zhang X., Chen T., Li J. (2019). Discovering Vulnerabilities in COTS IoT Devices through Blackbox Fuzzing Web Management Interface. Secur. Commun. Netw..

[22] Bezawada B., Ray I., Ray I. (2021). Behavioral fingerprinting of Internet-of-Things devices. Wiley Interdiscip. Rev. Data Min. Knowl. Discov..

[23] Ghazanfar S., Hussain F., Rehman A.U., Fayyaz U.U., Shahzad F., Shah G.A. Iot-flock: An open-source framework for iot traffic generation. Proceedings of the 2020 International Conference on Emerging Trends in Smart Technologies (ICETST).

[24] Khalil U., Ahmad A., Abdel-Aty A.H., Elhoseny M., El-Soud M.W.A., Zeshan F. (2021). Identification of trusted IoT devices for secure delegation. Comput. Electr. Eng..

[25] Xiong W., Lagerström R. (2019). Threat modeling–A systematic literature review. Comput. Secur..

[26] Li X., Zhang D., Wu B. Detection method of phishing email based on persuasion principle. Proceedings of the 2020 IEEE 4th Information Technology, Networking, Electronic and Automation Control Conference (ITNEC).

[27] Ferreira A., Coventry L., Lenzini G. (2015). Principles of persuasion in social engineering and their use in phishing. Proceedings of the International Conference on Human Aspects of Information Security, Privacy, and Trust.

[28] Nishikawa H., Yamamoto T., Harsham B., Wang Y., Uehara K., Hori C., Iwasaki A., Kawauchi K., Nishigaki M. Analysis of Malicious Email Detection using Cialdini’s Principles. Proceedings of the 2020 15th Asia Joint Conference on Information Security (AsiaJCIS).

[29] Sonowal G. (2020). Phishing Email Detection Based on Binary Search Feature Selection. SN Comput. Sci..

[30] Sahingoz O.K., Buber E., Demir O., Diri B. (2019). Machine learning based phishing detection from URLs. Expert Syst. Appl..

[31] Fang Y., Zhang C., Huang C., Liu L., Yang Y. (2019). Phishing email detection using improved RCNN model with multilevel vectors and attention mechanism. IEEE Access.

[32] Helmi R.A.A., Ren C.S., Jamal A., Abdullah M.I. Email Anti-Phishing Detection Application. Proceedings of the 2019 IEEE 9th International Conference on System Engineering and Technology (ICSET).

[33] Venkatraman S., Surendiran B., Kumar P.A.R. (2020). Spam e-mail classification for the Internet of Things environment using semantic similarity approach. J. Supercomput..

[34] Gupta B.B., Tewari A., Jain A.K., Agrawal D.P. (2017). Fighting against phishing attacks: State of the art and future challenges. Neural Comput. Appl..

[35] Li W., Meng W., Tan Z., Xiang Y. (2019). Design of multi-view based email classification for IoT systems via semi-supervised learning. J. Netw. Comput. Appl..

[36] Aleroud A., Abu-Shanab E., Al-Aiad A., Alshboul Y. (2020). An examination of susceptibility to spear phishing cyber attacks in non-English speaking communities. J. Inf. Secur. Appl..

[37] Kwak Y., Lee S., Damiano A., Vishwanath A. (2020). Why do users not report spear phishing emails?. Telemat. Inform..

[38] Suri R.K., Tomar D.S., Sahu D.R. (2012). An approach to perceive tabnabbing attack. Int. J. Sci. Technol. Res..

[39] Lim W.H., Liew W.F., Lum C.Y., Lee S.F. Phishing Security: Attack, Detection, and Prevention Mechanisms. Proceedings of the International Conference on Digital Transformation and Applications (ICDXA) 2020.

[40] Moul K.A. Avoid Phishing Traps. Proceedings of the 2019 ACM SIGUCCS Annual Conference.

[41] Hong J. (2012). The state of phishing attacks. Commun. ACM.

[42] Cova M., Kruegel C., Vigna G. (2008). There Is No Free Phish: An Analysis of “Free” and Live Phishing Kits. WOOT.

[43] Han X., Kheir N., Balzarotti D. Phisheye: Live monitoring of sandboxed phishing kits. Proceedings of the 2016 ACM SIGSAC Conference on Computer and Communications Security.

[44] Thomas K., Li F., Zand A., Barrett J., Ranieri J., Invernizzi L., Markov Y., Comanescu O., Eranti V., Moscicki A. Data breaches, phishing, or malware? Understanding the risks of stolen credentials. Proceedings of the 2017 ACM SIGSAC Conference on Computer and Communications Security.

[45] Cova M., Kruegel C., Vigna G. Detection and analysis of drive-by-download attacks and malicious JavaScript code. Proceedings of the 19th International Conference on World Wide Web.

[46] Common Vulnerabilities and Exposures (CVE). http://cve.mitre.org/.

[47] Frei S., Duebendorfer T., Ollmann G., May M. (2008). Understanding the Web Browser Threat: Examination of Vulnerable Online Web Browser Populations and the“Insecurity Iceberg”.

[48] Qin T., Burgoon J.K. An investigation of heuristics of human judgment in detecting deception and potential implications in countering social engineering. Proceedings of the 2007 IEEE Intelligence and Security Informatics.

[49] Mitnick K.D., Simon W.L. (2003). The Art of Deception: Controlling the Human Element of Security.

[50] SAE International (2016). Taxonomy and Definitions for Terms Related to Driving Automation Systems for On-Road Motor Vehicles.

[51] The 5 Levels of Autonomous Vehicles. https://www.truecar.com/blog/5-levels-autonomous-vehicles/.

[52] Fleetwood J. (2017). Public health, ethics, and autonomous vehicles. Am. J. Public Health.

[53] The Path to Autonomous Driving. https://www.bmw.com/en/automotive-life/autonomous-driving.html.

[54] Waymo Safety Report. https://storage.googleapis.com/sdc-prod/v1/safety-report/2020-09-waymo-safety-report.pdf.

[55] Vousden M. Level 5 Fully Self-Driving Cars Not Due Anytime Soon. https://www.just-auto.com/comment/level-5-fully-self-driving-cars-not-due-anytime-soon_id196671.aspx.

[56] Cho K.T., Shin K.G. Fingerprinting electronic control units for vehicle intrusion detection. Proceedings of the 25th {USENIX} Security Symposium ({USENIX} Security 16).

[57] foreseeti Automated Threat Modeling and Attack Simulations. https://www.foreseeti.com/.

[58] An Automated Threat Modeling Solution that Secures and Scales the Enterprise Software Development Life Cycle. https://threatmodeler.com/.

[59] Howell J., Kess B., Baldwin Microsoft Threat Modeling Tool. https://docs.microsoft.com/en-us/azure/security/develop/threat-modeling-tool.

[60] Shevchenko N., Chick T.A., O’Riordan P., Scanlon T.P., Woody C. (2018). Threat Modeling: A Summary of Available Methods.

[61] Scandariato R., Wuyts K., Joosen W. (2015). A descriptive study of Microsoft’s threat modeling technique. Requir. Eng..

[62] Parkinson S., Ward P., Wilson K., Miller J. (2017). Cyber threats facing autonomous and connected vehicles: Future challenges. IEEE Trans. Intell. Transp. Syst..

[63] Jacobsson A., Boldt M., Carlsson B. (2016). A risk analysis of a smart home automation system. Future Gener. Comput. Syst..

[64] Ashraf Q.M., Habaebi M.H. (2015). Autonomic schemes for threat mitigation in Internet of Things. J. Netw. Comput. Appl..

[65] Yan C., Xu W., Liu J. (2016). Can you trust autonomous vehicles: Contactless attacks against sensors of self-driving vehicle. DEF CON.

